# Differential accumulation of enterococci and arsenic in pelagic *Sargassum* and seagrass wrack on South Florida beaches

**DOI:** 10.1007/s10661-025-14888-5

**Published:** 2026-01-14

**Authors:** Afeefa A. Abdool-Ghany, Ayaaz Amirali, Rivka Reiner, Sofia Hoffman, Isabela Tavarez, Matthew Roca, Jiayu Li, Helena Solo-Gabriele

**Affiliations:** 1 Department of Chemical, Environmental and Materials Engineering, College of Engineering, University of Miami, Coral Gables, Florida USA; 2 Department of Mechanical and Aerospace Engineering, College of Engineering, University of Miami, Coral Gables, Florida USA

**Keywords:** Bacteria, Seaweed, Metals, Sand, Coastal beach management

## Abstract

Wrack, composed of organic debris like seagrass and the brown macroalga, *Sargassum*, accumulates on beaches and when present in overwhelming quantities can impact ecosystem function and public health through microbial contamination, trace element accumulation, and toxic gas emissions. Although studies have started to evaluate the impacts to the microbial quality from different types of wrack, few evaluate the impacts of trace elements. This study assessed enterococci and arsenic levels in beach environments across five South Florida beaches with varying wrack types (*Sargassum* vs. seagrass) and management practices. Enterococci levels did not significantly differ between wrack types (*p* = 0.30), with a maximum of 9,600 CFU/g. However, *Sargassum* exhibited significantly higher arsenic concentrations (up to 64.3 mg/kg) compared to seagrass (2.18 mg/kg) (*p* < 0.001). In sand, arsenic levels were statistically higher (4.92 mg/kg) when *Sargassum* was managed through integration. These findings emphasize the need to consider wrack composition when assessing arsenic impacts and can inform beach management strategies to minimize environmental and public health risks associated with excessively large *Sargassum* strandings.

**Supplementary Information:**

The online version contains supplementary material available at 10.1007/s10661-025-14888-5.

## Introduction

Beach wrack, organic plant and algal matter stranded on beach shores through the action of tides, wind, and waves (Graca et al., 2022), has been increasing worldwide due to the impacts of climate change and eutrophication (Lapointe et al., 2018; Robledo et al., 2021; Theirlynck et al., 2023; Wang et al., 2019). Seagrasses and *Sargassum* are two main contributors to wrack at beaches (Vázquez-Delfín et al., 2021), especially in Florida, US (Dierssen et al., 2015; Hatt et al., 2024). Seagrasses are marine flowering plants, possessing leaves, flowers, seeds, roots, and connective tissue (Nordlund et al., 2018) that grow submerged under water in soft sediment beds near coastlines. *Sargassum* is a genus of brown macroalgae first described by C. Agardh in 1820. Among the several hundreds of *Sargassum* species (Stiger-Pouvreau et al., 2023), only two, *S. natans* and *S. fluitans*, with morphotypes I, VIII, and III respectively, are holopelagic or free-floating throughout their life cycle (Theirlynck et al., 2023). In this paper, the term “*Sargassum*” refers to these two holopelagic species.

The western North Atlantic has been long recognized as an area of massive holopelagic *Sargassum* accumulations (Parr et al., 1939), with distributions throughout the Sargasso Sea, Caribbean Sea, and the Gulf Stream and the Gulf’s Loop Current (Lapointe et al., 2025). However, more recently, since 2011, a new area of *Sargassum* accumulation has been identified as the Great Atlantic *Sargassum* Belt (Wang et al., 2019), which serves as an added source of wrack primarily to coastal communities that surround the North Atlantic Ocean (Rodríguez-Martínez et al., 2025) and with impacts also noted in southern Brazil (Silva et al., 2023) in the Southern Atlantic. With changing climate, *Sargassum* is anticipated to increase in biomass (Tomenchok et al., 2021), making its impacts far-reaching globally. In “normal” stranding scenarios, *Sargassum* plays an important role in maintaining coastal ecosystem health (Lapointe et al., 2025). However, when the influxes are very large, coastal systems are overwhelmed, threatening ecosystems, fisheries, tourism, and public health (Chávez et al., 2020; Ofori & Rouleau, 2021; Rodríguez-Martínez et al., 2025).

When excessive, the wrack is managed at beaches using different strategies (Alleyne et al., 2025; Hamel et al., 2024; Saba et al., 2025). In the Yucatan Peninsula of Mexico, the material is removed as it approaches the shore and after stranding (Chávez et al., 2020). This practice of harvesting the *Sargassum* in-water is not permitted in the southeastern US waters, however, because *Sargassum* is protected as a critical habitat for fisheries and marine ecosystems (National Oceanic and Atmospheric Administration (NOAA), 2025). As a result, in South Florida, wrack is managed at beaches once it strands on-shore using burial or integration (mixing into sand). When quantities are excessive, the *Sargassum* is hauled off-site to landfills or to compost facilities (Abdool-Ghany et al., 2023a, 2023b).

The impacts of beach wrack when in excess are far-reaching, including contamination of the beach environment. Studies have started to document the contribution of wrack towards the proliferation of fecal indicator bacteria, enterococci (Abdool-Ghany et al., 2022). However, there is a new concern which has not gained much attention until recently: the impacts of metals, in particular arsenic, due to the ability of *Sargassum* to hyperaccumulate this metalloid. *Sargassum* species and most brown macroalgae are known to bioaccumulate trace metals from their surroundings, especially arsenic (Cipolloni et al., 2022; Dassié et al., 2021; Devault et al., 2020, 2021; McGillicuddy et al., 2023). Brown macroalgae bioaccumulate arsenic through passive and active uptake mechanisms. Arsenate (As^5^⁺), structurally similar to phosphate, is absorbed via phosphate transporters, while arsenite (As^3^⁺) diffuses directly across cell membranes. Within the algal tissues, arsenic can be transformed into organic forms like arsenosugars, which may become environmentally reactive when the algae decompose, potentially leaching toxic inorganic arsenic into beach sand and coastal waters.

The concern about arsenic is driven by its toxicity at low levels. Regulatory guidelines for arsenic are dependent upon the intended use of a product. The US EPA has established a guideline level of 75 mg/kg for land application of sewage sludge. States such as Florida have established Soil Cleanup Target Levels (SCTL), which provide guideline levels for recycled products such as mulches and compost for excessive potential risks in residential settings (2.1 mg/kg) and in industrial settings (12 mg/kg) (Florida Department of Environmental Protection, 2013).

Due to the toxicity of arsenic at low concentrations, studies have been initiated to evaluate arsenic levels in *Sargassum.* Studies have evaluated the levels of arsenic in pelagic *Sargassum* (Alleyne et al., 2023; Bam et al., 2022; Cipolloni et al., 2022 and others) at levels of several 100’s of mg/kg (Lapointe et al., 2025; McGillicuddy et al., 2023). Fewer have evaluated arsenic levels in stranded *Sargassum* (Devault et al., 2022; Liranzo-Gómez et al., 2023). Olguin-Maciel et al. (2022) documented that leachate generated during the decomposition of stranded *Sargassum* represents a risk of arsenic contamination. In addition to leaching during stranding, the levels of stranded *Sargassum* tend to be lower than observed among off-shore free-floating *Sargassum*, suggesting that arsenic depurates as it moves on-shore (Gobert et al., 2022). Even with lower levels at the shore, studies that evaluated stranded *Sargassum* have found the arsenic content of stranded *Sargassum* above those allowed by international guidelines for agriculture and animal feed (Ortega-Flores et al., 2023). In the nearshore waters of Miami, FL, levels of As in *Sargassum* have been measured at 15.1 to 120 mg/kg (Hatt et al., 2024). Along the coast of Corpus Christi, TX, levels were between 5.9 and 7.2 mg/kg (Sembera et al., 2018), suggesting that even at the lower bounds, the concentration of As in *Sargassum* consistently exceeds risk-based thresholds.

Among studies that have evaluated stranded *Sargassum*, none have systematically evaluated the levels of arsenic at beaches (in water, sand, and *Sargassum*) in the context of wrack composition and beach management practices. There is currently a gap in understanding about the impacts of decomposing wrack (*Sargassum* versus seagrass) on the levels of arsenic at beaches. Very limited information is available on the potential impacts of beach management practices in ameliorating the negative impacts of excessively large wrack strandings. To address this information gap, this study aimed to evaluate the impacts of stranded wrack on enterococci and arsenic levels in the beach environment in the context of different beach management practices and different wrack types. Enterococci were included in addition to arsenic as *Sargassum* also impacts the levels of these fecal indicators and it is the only environmental parameter currently used to assess beach quality in the US. Levels of enterococci and arsenic in beach water, sand, and wrack were documented for five beaches. The analysis documented the composition of the wrack between *Sargassum* and seagrasses and documented beach wrack management practices. The knowledge gained from this study can be used to develop management strategies for beach wrack in terms of its composition (*Sargassum* versus seagrass) and provide information about the potential effectiveness of grooming practices to decrease exposures to enterococci and arsenic at beaches impacted by the increasing volumes of *Sargassum*.

## Methods

### Site description and wrack management practices

**Fig. 1:**
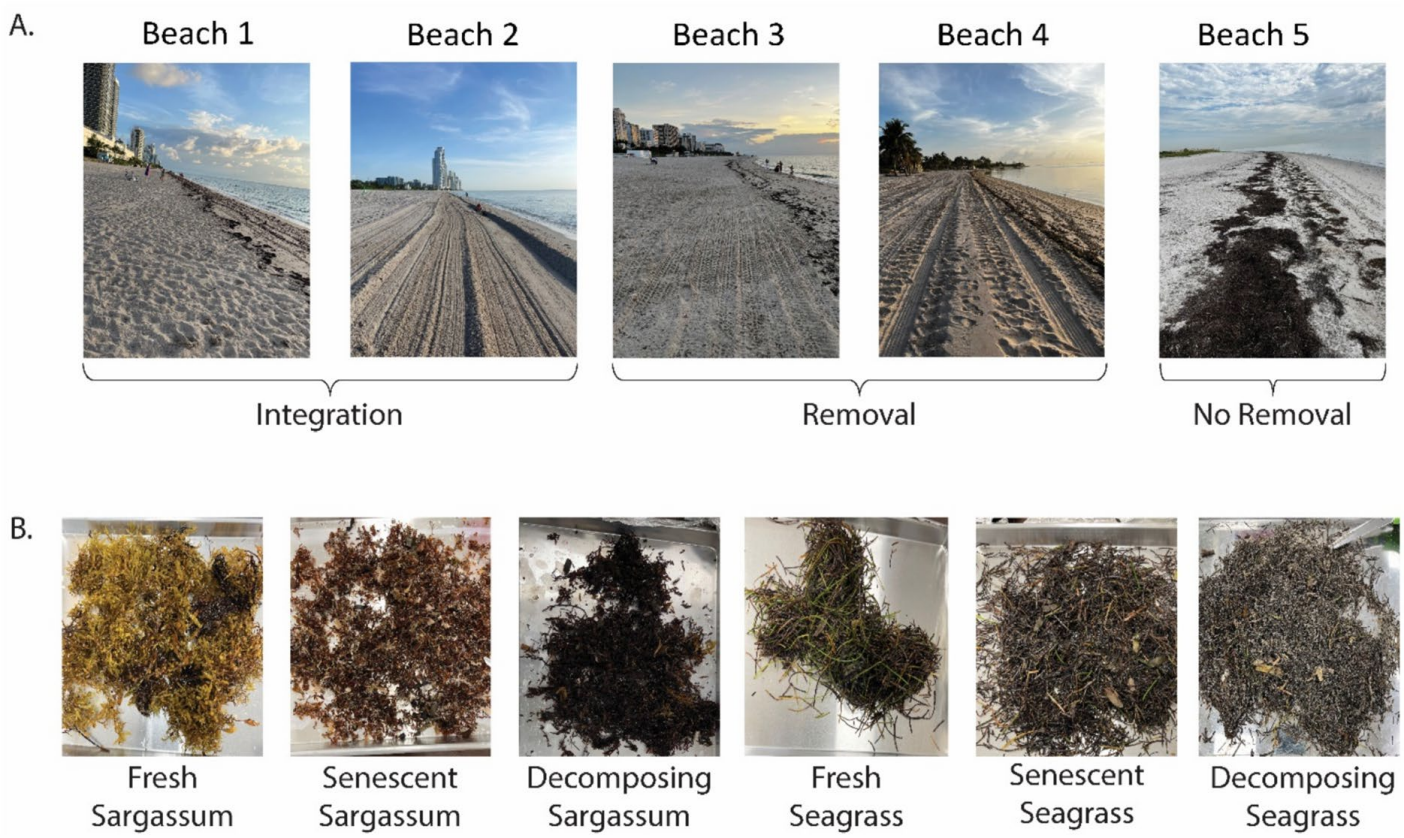
**A** Photographs illustrating beach zones categorized by *Sargassum* and seagrass management strategies: integration (in-place decomposition), no removal (unmanaged accumulation), and removal (manual or mechanical extraction). **B** Images representing the stages of *Sargassum* and seagrass degradation: fresh (recently deposited), senescent (partially decayed), and decomposing (advanced breakdown)

The five beaches evaluated were located in Southeast Florida (identified as B1 through B5); four were located in Miami-Dade County, and one was located in Broward County. The GPS coordinates are provided in the supplemental material. The beaches varied in exposure and morphology, with open coastlines (B2, B3, B4) and semi-enclosed settings (B1, B5), which may affect wrack retention and decomposition rates. The selection of each beach was informed by its wrack management approach, which included no removal (one beach), integration (two beaches), and removal (two beaches). Wrack was removed from the two beaches using a beach chariot (Surf Rake by Barber) connected to a tractor. The chariot removes the wrack by lifting the wrack over a conveyor belt with perforations that allow the sand to fall back onto the beach while capturing the wrack. For the two beaches that managed wrack through integration, two different integration processes were used. The first integration method utilized a “pull bar” which was composed of a large wooden pole attached to a tractor that was dragged across the beach sand at the wrack line, pressing the wrack into the sand. The second integration method consisted of a rear-mounted blader connected to a tractor (see Supplement, Figure S-2 for equipment photos). The rear-mounted blader consisted of an attached blade that cut wrack into smaller pieces and then mixed and smoothed it into the underlying sand. The practice of “no removal” allowed for the accumulation of wrack along various wrack lines, letting the wrack dry onshore and occasionally be carried back into the water depending upon the stranding location and tide (Fig. 1, Table 1).

**Table 1:** Beach management and wrack characteristics for the study beaches

Beach ID	Wrack Management Style	Amount of Wrack by Sampling Date^a^			Wrack Composition by Sampling Date		
		Jul. 6	Aug. 3	Sep. 7	Jul. 6	Aug. 3	Sep. 7
B1	Integration: Pull Bar	H	M	L	*Sargassum*	*Sargassum*	*Sargassum*
B2	Integration: Blader	L	L	L	*Sargassum*	*Sargassum*^*b*^	*Sargassum*
B3	Removal: Beach Chariot	H	L	L	*Sargassum*^*a*^	*Sargassum*^*b*^	*Sargassum*
B4	Removal: Beach Chariot	M	H	L	Seagrass	*Sargassum*	Seagrass
B5	No Removal	L	H	H	Seagrass	*Sargassum*^*b*^	Seagrass

^a^The amount of wrack was visually categorized as low (< 25% beach coverage), medium (25 to 50%) or high (> 50%) based upon photographic documentation and field observations. See Supplement Figure S-1 for photos

^b^The sample was composed primarily of *Sargassum* with small amounts of seagrass estimated at 95% *Sargassum* and 5% seagrass mix

### Field data collection

Field data collection included the temperature of the water, sand, and wrack (MT Raytek® laser thermometer) plus water pH, salinity, and turbidity (Xylem ProDSS Water Quality Sonde). Ambient weather conditions (air temperature and humidity) were obtained from the iPhone weather app, which sources data from the National Weather Service and the National Oceanic and Atmospheric Administration for the location. On average, the water pH, salinity, and turbidity were 8.1, 33.6 psu, and 4.8 ntu (see Table S-2 for details). Air temperature and humidity at the time of sample collection were 27.2 °C and 83%, respectively. Average water, sand, and wrack temperatures were 29.9 °C, 26.9 °C, and 27.2 °C, respectively. The amount of wrack was visually categorized as low (L: < 25% beach coverage), medium (M: 25–50%), or high (H: > 50%) based on photographic documentation and field observations (Supplement, Figure S-1). The wrack samples were predominantly composed of either *Sargassum* or seagrass and were further categorized in the lab as fresh, senescent, or decomposing based on moisture content. At the laboratory, the wrack samples were placed on a sterile tray and photographed. These photographs were used to confirm the wrack composition (usually either predominantly *Sargassum* or seagrass). The *Sargassum* was characterized as either *S. natans I*, *S. natans VIII*, or *S. fluitans III* (Iporac et al., 2022). Photographs and additional details about the field data are available in the supplemental text.

### Sample collection

Samples were collected at each of the five beaches during the early morning hours (near sunrise) on three different dates (July 6, 2023, August 3, 2023, and September 7, 2023). The early morning sampling time was important for beaches that practiced daily wrack removal, as it facilitated the collection of wrack that may have stranded overnight. Two sampling teams were deployed to facilitate collection prior to beach grooming activities. The five-beach sampling effort was conducted on three different dates during the summer beach season to provide replication that could account for changing conditions during the summer months. At each beach, water, sand, and wrack samples were collected along a transect (line perpendicular to the water’s edge). On each sampling date, one water sample (0.5 L), three sand samples (supratidal, under-wrack, and bladed when applicable, 1 kg each), and one wrack sample (1 L volume picked from various locations on the stranding line) were collected per beach, resulting in a total of 15 water samples, 45 sand samples, and 15 wrack samples across the three sampling dates. The water samples were collected in ankle-deep water by scooping the water surface with a Whirlpak bag from an area upstream and undisturbed by the sampler. Similarly, for each beach and each sampling day, three sets of sand samples were collected from the upper 2 cm of the sand surface using a sterile spoon. Sand samples were collected above the high tide wrack line, called “supratidal” sand; another was collected below the wrack, called “under” sand, and for the beach that bladed wrack, a “bladed” sample was collected when observed. Bladed samples are produced by machinery that shreds the *Sargassum* into about 2 cm pieces and mixes it with the upper layers of the sand, resulting in the integration of *Sargassum* into the sand. Wrack was placed into a sterile Whirlpak bag using clean gloves. During one of the sampling days for Beach 1, two wrack lines were observed, and two wrack samples along with the corresponding sand “under” samples were collected. Although the samples from each wrack line were analyzed separately, the results from this one sampling day from Beach 1 were averaged together for data analysis purposes. Upon collection, all samples were immediately placed into a cooler with ice and transported back to the University of Miami laboratory for processing.

### Enumeration of Enterococci

Enterococci were processed immediately upon arrival at the laboratory (within 2.5 h of collection). Upon arrival at the laboratory, water samples were homogenized by shaking and split two ways, one for enterococci analyses and another for arsenic analyses. For sand and wrack, samples were homogenized by mixing with a sterile spoon and split three ways: for the analysis of enterococci, arsenic, and moisture content (MC). MC was used to normalize the enterococci counts by mass of dry sand or mass of dry wrack. In addition, MC was used to categorize the wrack samples composed predominantly of *Sargassum* into fresh (MC ≥ 75%), senescent (74% < MC < 55%), and decomposing (MC ≤ 54%) as per prior studies (Abdool-Ghany et al., 2022). Moisture content was determined gravimetrically (110 °C after 24 h).

Enterococci measurements followed standard membrane filtration protocols (U.S. EPA 2014, Method 1600). The process involved filtering a known volume of sample through a sterile membrane filter (0.45-μm pore size mixed cellulose filters, Pall Industries GN-6) and placement of the filter onto Enterococcus indoxyl-β-D-glucoside (mEI) agar (Aquaplates). Filtration volumes included 100 mL for water, 2 mL plus 20 mL for sand eluates, and 1 mL and 10 mL for wrack eluates. Once placed on the agar, filters were incubated at 41 ± 0.5 °C for a duration of 24 h. After incubation, colonies were counted. Any colonies that displayed a blue color or presented a blue halo were counted as positive.

Enumeration of sand and wrack required the elution of microbes from the sediment (Boehm et al., 2009) or wrack (Abdool-Ghany et al., 2022) into a sterile phosphate-buffered saline solution. In brief, for sand, a measured weight of sediment (about 10 g) was placed into a sterile 100 mL bottle. Then, 100 mL of sterile phosphate-buffered saline (PBS) was added, and the bottle was shaken for 2 min. The solution was allowed to settle for 2 min before aliquots (2 mL and 20 mL) of the eluate were drawn and filtered. For wrack, 200 mL of sterile PBS was added to a measured weight (about 10 g) of aseptically cut seaweed placed within a sterile Whirlpak bag. The bacteria were eluted by rubbing the bag between the thumb and fingertips for 2 min, followed by a 2-min settling period. The eluate was drawn (1 mL and 10 mL) and filtered through the membranes. Since there were 2 dilutions used for sand and wrack, the average of the two values was used for subsequent data analysis. Plate results consistently showed lower counts for the plates receiving the smaller volume of eluate.

### Arsenic analysis

Water sample splits for arsenic analysis were placed into new pre-acid washed 250 mL high-density polyethylene (HDPE) bottles containing 1 mL of concentrated nitric acid. For sand and wrack, splits for arsenic analysis were placed into new pre-acid washed 60 mL glass wide-mouth jars. Once samples were placed in their respective containers, they were refrigerated and then transported in a cooler with frozen ice-packs to the analytical laboratory (Florida Spectrum Environmental Services) within 2 days of collection and analyzed within 7 days of collection. Standardized methods were used for the digestion of samples. For water, samples were digested using concentrated nitric and hydrochloric acid (US EPA, 1992, Method 3010). For sand and wrack, samples were digested using concentrated nitric and hydrochloric acid and hydrogen peroxide (US EPA, 1996, Method 3050). Inductively Coupled Plasma, Atomic Emission Spectroscopy (ICP-AES, Agilent Technologies Model 5110) was used to analyze the digestates (US EPA, 1994, Method 200.7 for water; US EPA, 2018, Method 6010D for sand and wrack). At Florida Spectrum, each sample batch included a blank, a laboratory control standard (LCS), a matrix spike (MS), and an MS duplicate. All blanks were below the detection limit for water (< 10 µg/L) and for solids (< 0.5 mg/kg). The LCS were within 101% of the arsenic concentration target for both water and solid samples. The MS and MS-duplicates showed 100.3% and 99.4% arsenic recoveries for the water and solid samples, respectively. The reproducibility of the MS and MS-duplicates was high, within 1% within batches and within 3% between batches. The sample detection limit for water was 30 µg/L due to the need to dilute the samples because of the high salt content. The sample detection limits for sand and wrack were 0.2 mg/kg and 0.9 mg/kg, on average.

### Statistical analysis

The Shapiro–Wilks test was used to assess the normality of the enterococci and arsenic data. The results of this analysis found that neither the enterococci nor arsenic concentrations were normally distributed; thus, prompting the use of non-parametric analyses. The Kruskal–Wallis H test was used to assess statistical differences in the enterococci and arsenic concentrations across the diverse types of samples collected as well as the various beaches sampled. The Dunn’s test was used for pairwise comparisons among sample categories. The one-sample Wilcoxon signed rank test was used to evaluate potential outliers. Among these statistical tests, the comparison of means with p-values less than 0.05 was considered statistically different.

## Results

**Fig. 2:**
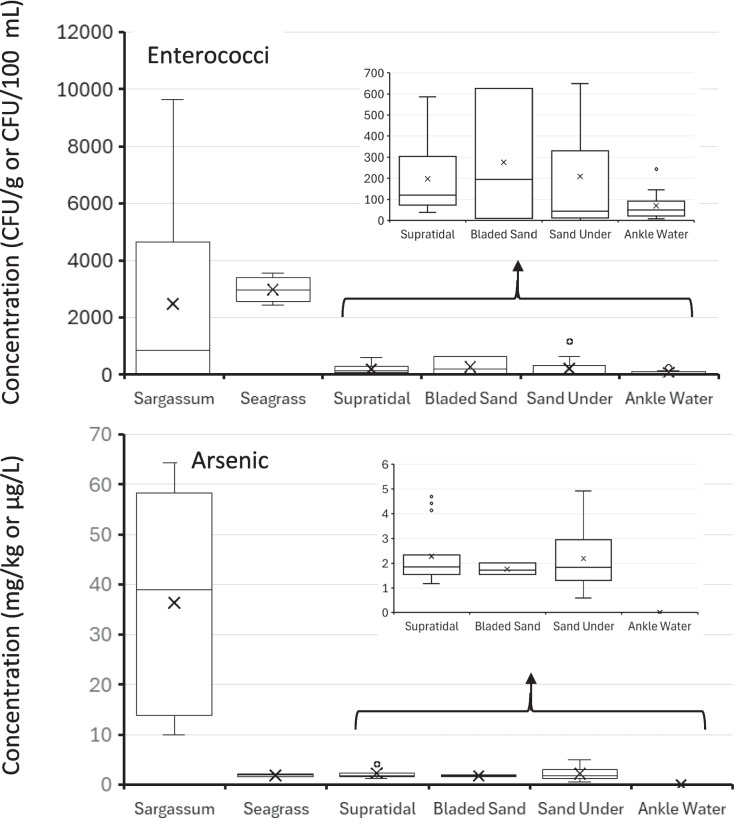
Results consolidated across the five sample beaches. The top panel shows enterococci in *Sargassum*, seagrass, sands (supratidal, bladed, and under) (CFU/g) and in ankle-deep water (CFU/100 mL). The bottom panel depicts arsenic concentrations within the same *Sargassum*, seagrass, and sand samples (mg/kg) and within the ankle-deep water (µg/L)

Collective results from all sampling events show that wrack (both *Sargassum* and seagrass) supports elevated levels of enterococci, whereas only the *Sargassum* component of the wrack supports elevated levels of arsenic (Fig. 2). In addition to the composition of the wrack, *Sargassum* beach management style appears to also play a role in arsenic levels in sand.

### Impact of management style on beach quality

**Fig. 3:**
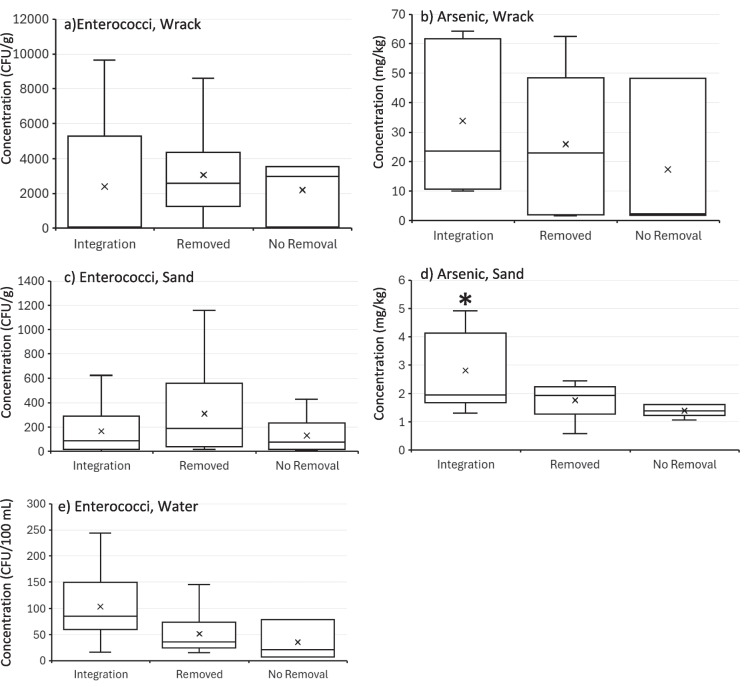
Comparison of enterococci and arsenic concentration in wrack, sand, and water from all five beach sites by beach management type (integration, removal, and no removal). Panels **a** and **b**, concentrations in wrack, combined *Sargassum* and seagrass. Panels c and **d**, concentrations in sand from all three sand zones, and Panel **e**, concentration of enterococci in water. Left panels emphasize enterococci concentrations and right panels emphasize the arsenic concentrations. Arsenic not shown for water because all samples were below the detection limit of 30 µg/L. The “*” symbol in panel d emphasizes the statistically different levels of arsenic in sand that had undergone integration

For enterococci, no significant differences were observed between management style and enterococci concentrations in wrack (*p* = 0.65), sand (*p* = 0.42), nor water (*p* = 0.17) (Fig. 3, panels a, c, and e). For arsenic, no significant differences were observed between management style and arsenic concentration in the water as all values were below the detection limit. In addition, no significant differences were observed between management style and arsenic concentration in the wrack (*p* = 0.56, Fig. 3 panel b). However, management style was statistically significantly associated with arsenic concentrations in the sand (*p* < 0.001, Fig. 3, panel d). Beaches that integrated *Sargassum* showed higher arsenic concentrations in sand compared to beaches that removed or did not remove *Sargassum*. Specifically, beaches with integration (e.g., Beach 1, Beach 2) had an average arsenic concentration of 2.75 mg/kg, significantly higher than the no removal sites (1.39 mg/kg, *p* < 0.001) (Table 2). The 2.75 mg/kg value for beaches that integrate *Sargassum* slightly exceeds Florida’s residential Soil Cleanup Target Level (SCTL) of 2.1 mg/kg. Beach 1 (integration by pull bar) had the highest arsenic concentration (4.31 mg/kg) compared to others, including Beach 3 (removal) and Beach 5 (no removal), which had lower levels (1.39 mg/kg) (Table 3). These findings suggest that management strategies involving integration may increase arsenic retention in beach sand, though differences could also be partially driven by wrack type and accumulation levels at each site.

**Table 2:** Summary of enterococci and arsenic concentrations by beach site and sampling day. Values bolded and not within the parenthesis correspond to averages. The averages for each beach from sample collection conducted on July 6, August 3, and September 7 are listed in the first 5 rows. Values listed under the next three rows under the B1-B5 categories correspond to the averages for the sampling day from all five beaches (B1 to B5) for the corresponding sampling day listed. Overall corresponds to the averages for all five beaches for all three sampling days. Values in parenthesis correspond to the number of samples analyzed (listed first) and the standard deviation of the results (listed second)

Category	Enterococci								Arsenic							
	Water (CFU/100 mL)^d^	Sand (CFU/g)				Wrack (CFU/g)			Water (µg/L)	Sand (mg/kg)^c^				Wrack (mg/kg)		
		Supra	Under	Bladed	All Sand	Seagrass	*Sargassum*	All Wrack		Supra	Under	Bladed	All Sand	Seagrass	*Sargassum*	All Wrack
B1	**81.7** (3)(8.6)	**221** (3)(138)	**139** (3)(186)		**180** (6)(153)		**2075** (3)(2816)	**2075** (3)(2816)	ND^a^ (3)(NA^b^)	**4.41** (3)(0.28)	**4.21** (3)(0.66)		**4.31** (6)(0.47)		**35.0** (3)(25.9)	**35.0** (3)(26.9)
B2	**126** (3)(114)	**158** (3)(128)	**77.6** (3)(132)	**276** (3)(316)	**171** (9)(202)		**3236** (3)(5548)	**3236** (3)(5548)	ND (3)(NA)	**1.60** (3)(0.28)	**1.77** (3)(0.12)	**1.76** (3)(0.24)	**1.71** (9)(0.21)		**32.7** (3)(28.1)	**32.7** (3)(28.1)
B3	**31.3** (3)(18)	**142** (3)(116)	**232** (3)(360)		**187** (6)(244)		**3424** (3)(4552)	**3424** (3)(4552)	ND (3)(NA)	**1.66** (3)(0.55)	**1.12** (3)(0.56)		**1.39** (6)(0.58)		**29.9** (3)(17.0)	**29.9** (3)(17.0)
B4	**71.7** (3)(64)	**240** (3)(301)	**631** (3)(469)		**435** (6)(413)	**2701** (2)(375)	**2742** (1)(NA)	**2715** (3)(267)	ND (3)(NA)	**2.15** (3)(0.20)	**2.13** (3)(0.28)		**2.14** (6)(0.22)	**1.79** (2)(0.34)	**62.5** (1)(NA)	**22.0** (3)(35.1)
B5	**35.7** (3)(38)	**231** (3)(175)	**27.6** (3)(23)		**129** (6)(158)	**3264** (2)(411)	**68.7** (1)(NA)	**2199** (3)(1867)	ND (3)(NA)	**1.55** (3)(0.11)	**1.23** (3)(0.15)		**1.39** (6)(0.21)	**1.98** (2)(0.29)	**48.2** (1)(NA)	**17.4** (3)(26.7)
B1-B5, July 6	**39.0** (5)(29)	**195** (5)(179)	**73.6** (5)(105)	**196** (1)(NA)	**140** (11)(146)	**2704** (2)(380)	**314** (3)(486)	**1270** (5)(1367)	ND (5)(NA)	**2.12** (5)(1.18)	**2.05** (5)(0.90)	**2.02** (1)(NA)	**2.08** (11)(0.94)	**1.66** (2)(0.16)	**33.3** (3)(10.4)	**20.7** (5)(18.8)
B1-B5 August 3	**106** (5)(92)	**156** (5)(121)	**343** (5)(242)	**625** (1)(NA)	**284** (11)(226)	NA	**5268** (5)(3988)	**5268** (5)(3988)	ND (5)(NA)	**2.16** (5)(1.29)	**2.18** (5)(1.21)	**1.73** (1)(NA)	**2.13** (11)(1.12)		**28.5** (5)(25.1)	**28.5** (5)(25.1)
B1-B5 September 7	**62.8** (5)(42)	**245** (5)(199)	**247** (5)(509)	**9.0** (1)(NA)	**224** (11)(353)	**3260** (2)(416)	**578** (3)(939)	**1651** (15)(1626)	ND (5)(NA)	**2.55** (5)(1.23)	**2.05** (5)(1.69)	**1.54** (1)(NA)	**2.23** (11)(1.39)	**2.11** (2)(0.11)	**53.7** (3)(16.2)	**33.1** (5)(30.5)
Overall	**69.3** (15)(63)	**198** (15)(161)	**210** (15)(320)	**276** (3)(316)	**211** (33)(253)	**2982** (4)(457)	**2638** (11)(3597)	**2730** (15)(3051)	ND (15)(NA^b^)	**2.27** (15)(1.16)	**2.19** (1)(1.24)	**1.76** (3)(0.24)	**2.19** (33)(1.14)	**1.88** (4)(0.28)	**36.7** (15)(21.2)	**27.4** (15)(24.0)

^a^ND = Not Detected. Detection limit for water samples was 30 µg/L. ^b^NA = Not Applicable. ^c^Health-based soil standard for Florida in residential areas is 2.1 mg/kg. ^d^Water quality guideline for beach advisories in Florida is 70 CFU/100 mL

**Table 3:** Summary of enterococci and arsenic concentrations by sample type, wrack decomposing status, and beach management style. Values listed in bold correspond to the averages for all five beaches for all three sampling dates. Values in parenthesis correspond to the number of samples analyzed within the category (first) and the standard deviation of the results (second)

Category	Enterococci (CFU/dry g or CFU/100 mL)			Arsenic (µg/g or µg/L)		
	Average	No	Std. dev	Average	No	Std. dev
Sample Type						
Seagrass	**2982**	(4)	(457)	**1.88**	(4)	(0.28)
*Sargassum*	**2638**	(11)	(3597)	**36.7**	(15)	(21.2)
Wrack, Overall	**2730**	(15)	(3051)	**27.4**	(15)	(24.0)
Supratidal Sand	**198**	(15)	(161)	**2.27**	(15)	(1.16)
Under Sand	**210**	(15)	(320)	**2.19**	(1)	(1.24)
Bladed Sand	**276**	(3)	(316)	**1.76**	(3)	(0.24)
Sand, Overall	**211**	(33)	(253)	**2.19**	(33)	(1.14)
Water	**69.3**	(15)	(63)	ND^a^	(15)	(NA^b^)
Wrack Decomposing Status						
All: Fresh	**1252**	(2)	(1673)	**24.9**	(2)	(33.0)
All: Senescent	**3556**	(7)	(3988)	**26.5**	(7)	(21.4)
All: Decomposing	**2258**	(6)	(2080)	**29.2**	(6)	(28.8)
Seagrass: Fresh	**2435**	(1)	(NA)	**1.55**	(1)	(NA)
Seagrass: Senescent	**2973**	(1)	(NA)	**1.77**	(1)	(NA)
Seagrass: Decomposing	**3260**	(2)	(416)	**2.11**	(2)	(0.1)
*Sargassum*: Fresh	**68.7**	(1)	(NA)	**48.2**	(1)	(NA)
*Sargassum*: Senescent	**3653**	(6)	(4360)	**30.7**	(6)	(20.2)
*Sargassum*: Decomposing	**1756**	(4)	(2479)	**42.8**	(4)	(25.5)
Management Style (*Sargassum* only)						
No Removal	**68.7**	(1)	(NA)	**48.2**	(1)	(NA)
Integration	**2655**	(6)	(3986)	**33.8**	(6)	(24.2)
Removal	**3253**	(4)	(3733)	**38.1**	(4)	(21.4)
Management Style (Seagrass only)						
No Removal	**2704**	(2)	(380)	**1.66**	(2)	(0.16)
Integration	NA	NA	NA	NA	NA	NA
Removal	**3260**	(4)	(416)	**2.11**	(4)	(0.11)
Management Style (Sand only)						
No Removal	**129**	(6)	(158)	**1.39**	(6)	(0.21)
Integration	**174**	(15)	(178)	**2.75**	(15)	(1.36)
Removal	**311**	(12)	(348)	**1.77**	(12)	(0.57)

^a^ND = Not Detected. All water samples were below the detection limit (30 µg/L)

^b^NA = Not Applicable

### Enterococci levels split by matrix and additional factors

#### Water

No significant differences in enterococci water levels were observed between beaches (*p* = 0.36).

#### Sand

No significant differences were observed in enterococci by beach (*p* > 0.17). For sand within beaches, no significant differences in enterococci levels were found between sand sample types (supratidal, sand under, and bladed).

#### Wrack

**Fig. 4:**
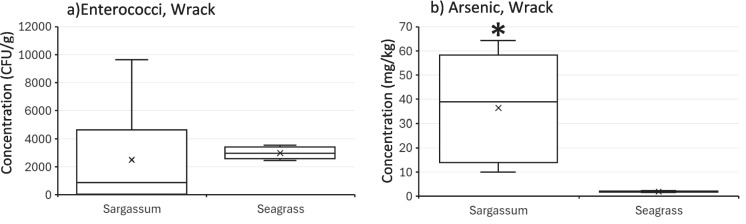
Comparison of *Sargassum* and seagrass. Left panel compares enterococci concentrations (CFU/g) and the right panel compares arsenic concentrations (mg/kg). The “*” symbol in panel b emphasizes the statistically different levels of arsenic in *Sargassum* relative to seagrass

No significant differences were observed in wrack enterococci concentrations across beaches (*p* = 0.46). Similarly, no significant differences were observed in enterococci concentrations among wrack types (*Sargassum* versus seagrass) (*p* = 0.21, Fig. 4, left panel). Moisture content in wrack (fresh, senescent, and decomposing) also did not significantly affect enterococci concentrations (*p* = 0.78).

### Arsenic levels split by matrix and additional factors

#### Water

Arsenic concentrations in water samples were all below detection limits (30 µg/L).

#### Sand

**Fig. 5:**
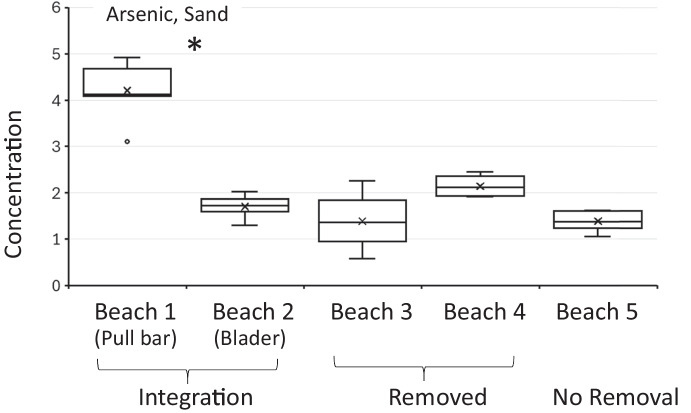
Arsenic concentrations in sand by beach emphasizing the *Sargassum* beach management practices

Significant differences were observed between Beach 1 (integrates by pull bar) and other beaches (e.g., B1 vs. B2 (*p* = 0.001), B3 (*p* = 0.0012), B4 (*p* = 0.0012), B5 (*p* = 0.0012)) (Fig. 5). No significant differences in arsenic concentrations were observed between sand sample types (supratidal, under, bladed; *p* = 0.83).

#### Wrack

*Sargassum* had significantly higher arsenic concentrations (36.4 mg/kg) compared to seagrass (1.9 mg/kg, p = 0.004) (Fig. 4). No statistical differences were observed among beach sites (*p* = 0.73) nor wrack decomposing statuses (*p* = 0.88).

## Discussion

### Enterococci concentrations in *Sargassum* and seagrass

Of interest was that although arsenic levels were statistically different between *Sargassum* and seagrass, the enterococci levels were not different statistically. This similarity in enterococci levels may be due to the similarity in which *Sargassum* and seagrass allow for the persistence and regrowth of enterococci. Studies have found associations between seaweed presence and enterococci levels (Anderson et al., 1997; Kelly et al., 2018). Abdool-Ghany et al. (2022) found that elevated levels of enterococci were confined to the beach wrack zone during periods of beach closures induced by the COVID-19 pandemic. They hypothesized that decomposing seaweed provides an additional substrate for enterococci to grow. The results from the current study support that both *Sargassum* and seagrass serve as possible sources of nutrients for growth, as observed for other macroalgae in freshwater environments (Badgley et al., 2011; Verhougstraete et al., 2010). The management practices of beach wrack, whether integration, removal, or no removal, did not significantly affect the persistence of enterococci on either substrate, indicating that other environmental factors such as moisture retention and organic matter could play a more significant role. It is also possible that the *Sargassum* and seagrass may facilitate persistence by retaining moisture and possible protection against UV light (Beckinghausen et al., 2014). Collectively, the results from the current study support that fecal indicator bacteria persist and grow indiscriminately on organic substrates that accumulate in coastal zones.

### Arsenic concentrations in *Sargassum* and seagrass

Arsenic was elevated in *Sargassum* compared to seagrass. Two mechanisms are believed to impact arsenic uptake by *Sargassum*. First, *Sargassum*, like many other brown seaweeds, accumulates arsenic and other trace elements (Alleyne et al., 2023; Fauser et al., 2013; Neff, 1997) through its cell-wall polysaccharides, particularly alginate and fucoidan (Ortega-Flores et al., 2022). Alginate contains carboxylic groups that serve as primary active sites for cation uptake, while sulfated fucoidans, with sulfonic acid groups, act as secondary active sites for sequestering divalent cations (Davis et al., 2004; Fourest & Volesky, 1997; Percival & McDowell, 1990). Seagrasses’ cell walls are composed of cellulose and sulfated polysaccharides (Pfeifer & Classen, 2020), which may not have the same sorptive ability as *Sargassum*. *Sargassum* has a documented capacity to uptake arsenic and other metals through sorption, whereas work is needed to evaluate whether seagrasses have the same capacity.

The second mechanism is due to the similarity between arsenate and primary nutrient phosphate. In nutrient-poor pelagic waters, *Sargassum* has evolved mechanisms for hyperaccumulating nutrients (Lapointe et al., 2025). The uptake of arsenate by *Sargassum* is correlated with areas of a high degree of phosphorus limitation (McGillicuddy et al., 2023). Unlike seagrasses, which absorb nutrients through their roots and leaves, *Sargassum* transports nutrients through its tissues by diffusion and through phosphate transporters, leading to distinct patterns of arsenic accumulation. This difference in nutrient buildup mechanisms results in significantly higher arsenic levels in *Sargassum* compared to seagrass, which does not uptake or sorb arsenic as effectively (Raize et al., 2004). As a result, wrack accumulations will vary substantially in arsenic content depending on the proportion of *Sargassum* versus seagrass. Management strategies that mix wrack into sand may amplify exposure risks, especially if *Sargassum* is dominant and highly concentrated in arsenic.

### Variability in arsenic concentrations due to management practices

In this study, the coefficient of variation (COV) for arsenic concentrations was highest at beaches practicing integration (49%), followed by removed (32%), and no removal (15%). The higher COV observed at sites utilizing integration suggests greater variability in arsenic levels, indicating inconsistent distribution within the samples. This variability is likely due to the mixing of sand and *Sargassum* during integration practices, which introduces a wider range of arsenic sources and concentrations. Such variability underscores the risks associated with integrating organic matter like *Sargassum* into beach sands, where arsenic from different sources may accumulate unevenly. In contrast, beaches where no removal was practiced exhibited the most consistent arsenic levels, with the lowest COV, reflecting more stable and predictable conditions. The removal approach demonstrated intermediate variability, likely reflecting reduced, but not eliminated, arsenic sources compared to integration. These findings highlight the influence of beach management practices on arsenic distribution and underscore the potential risks associated with integration, where mixing could enhance variability in contaminant exposure. Of significance was that the average arsenic concentration in sand was higher at the beaches that practiced integration. The mean sand arsenic concentration for these beaches (2.8 mg/kg) was slightly above the Florida SCTL (2.1 mg/kg). Compared to beaches that did not practice integration (1.8 mg/kg for removal and 1.4 mg/kg for no removal), the differences were not large, suggesting that beach sand does not retain arsenic to a great extent. The lack of sorption of arsenic by beach sand is consistent with studies that have found limited sorption under conditions of low organic and iron content (Fauser et al., 2013). This reinforces the concern that wrack type and biomass load may be confounding factors in assessing the effects of management alone. Quantifying wrack volume and composition more precisely in future studies will help separate these effects.

The beach that showed the highest sand arsenic concentrations (B1, 4.3 mg/kg) practiced integration by pull bar. The pull bar compacts the wrack into the sand, likely limiting the ability of rainwater to infiltrate, thereby resulting in less flushing through rainfall infiltration. There is also the possibility that the pull bar utilized by B1 could have been composed of a common wood preservative used for utility poles, known as chromated copper arsenate, which contains very high levels of arsenic (Dubey et al., 2007; Jones et al., 2019; Shibata et al., 2007). This highlights the need for further examination of the materials used in integration equipment and their potential contribution to arsenic contamination. Of interest was that the beach that practiced no removal (B5) had one of the lowest concentrations (1.4 mg/kg), lower than other beaches practicing removal, equal to one beach that practiced wrack removal and lower than another that also practiced wrack removal. It appears that wrack removal does not provide advantages over non-removal in terms of sand arsenic concentrations. Similar to the compaction hypothesis with the pull bar method of integration, it is possible that the equipment used to remove the arsenic from the beach can be compacting the sand, thereby limiting flushing by rainwater infiltration, or the equipment itself can also be contaminating the beach environment. More research is needed to evaluate these hypotheses and to confirm the relative sand arsenic levels between beaches.

## Limitations

Limitations of the study included the small number of beaches analyzed and the limited number of sampling events per beach, combined with variability in wrack composition between sites. This made it difficult to isolate the effect of management from the influence of *Sargassum* versus seagrass presence. The study was conducted during 2023, which was not a peak *Sargassum* bloom year. Arsenic concentrations and wrack volumes could be higher during high influx years, limiting the generalizability of the findings. The limited number of beaches sampled coupled with a lack of replication sampling during each of the sampling dates may have reduced the statistical power of the study, making it difficult to detect significant differences or trends across different locations or conditions. Another limitation may be that the selected beaches experience different stranding conditions, which could impact comparisons across management practices. A larger dataset would enhance the ability to draw more reliable conclusions and potentially uncover subtle variations in arsenic levels, moisture content, and other relationships between beach wrack and enterococci and arsenic levels. Future studies should incorporate replication and finer temporal resolution to capture variability. Additionally, a more extensive geographic range of sampling sites could provide insights into regional differences in *Sargassum* composition and decomposition dynamics, contributing to a more comprehensive understanding of its environmental impacts.

## Recommendations

Future studies should better define the decomposition state of the wrack upon collection. In this study, moisture content was used as the proxy. However, other wrack characteristics should be considered, including color, assessment of photosynthesis capacity (chlorophyll *a* and pheophytin), nutrient levels (nitrogen and phosphorus), and prior environmental conditions such as time since stranding and last wave inundation and prior rainfall. To further understand the decomposition of *Sargassum* wrack, we recommend mesocosm experiments designed to evaluate how different environmental conditions influence the breakdown of *Sargassum* and the release of arsenic and other trace elements. As *Sargassum* decomposes and dries out on beaches, it would be valuable to determine whether arsenic is released into surrounding media or remains bound within the biomass. These experiments could provide insights into whether the drying process leads to a loss of arsenic, potentially through desorption, leaching, or volatilization. Such findings would have significant implications for managing *Sargassum* wrack on shorelines, particularly in areas where arsenic contamination poses environmental or public health concerns.

Quantifying *Sargassum* biomass, such as volume, height, or percent coverage, should also be incorporated into future monitoring efforts. Wrack load may be an important factor in trace element accumulation in adjacent sand or water, and such data would improve the ability to model contaminant fluxes. In parallel, it is important to incorporate precipitation data, as arsenic mobility may increase during wet seasons due to greater leachate formation and infiltration. Nearshore water monitoring during brown tide events, particularly those linked to *Sargassum* decay, is also recommended. These events can possibly elevate arsenic and dissolved organic matter concentrations, warranting further study to evaluate potential impacts to swimmers and seafood consumers.

Finally, to support evidence-based beach management, future research should explore additional variables such as the materials used in grooming equipment (Kinzelman et al., 2004), including any components treated with arsenic-based preservatives or containing metals that could contribute to contamination. Establishing thresholds for *Sargassum* removal based on wrack type, arsenic concentration, and decomposition stage would help local governments make informed decisions about when and how to manage coastal wrack.

## Conclusions

The increasing accumulation of *Sargassum* on global coastlines presents significant environmental challenges, particularly concerning arsenic and bacteria dynamics and ecosystem health. *Sargassum’s* capacity to bioaccumulate trace elements, particularly arsenic, and its potential to influence microbial contamination raise concerns about the ecological and public health impacts of current beach management practices. The results from this study emphasize the importance of documenting wrack characteristics, particularly the distinction between *Sargassum* and seagrass, when evaluating beach management strategies targeting fecal indicator bacteria and arsenic levels. Enterococci were not sensitive to wrack type, but arsenic was. At the study beaches, those that managed wrack through integration had statistically higher arsenic concentrations in sand than those that did not remove wrack. Additional monitoring across more beaches and seasons is needed to validate these trends. Risk assessments should also be conducted to evaluate whether elevated arsenic in beach sand and wrack poses a health concern to beachgoers. This data can be used to inform thresholds for wrack removal to ensure that arsenic levels at beaches remain within acceptable concentrations.

## Supplementary Information

Below is the link to the electronic supplementary material.

ESM 1 (DOCX 7.07 MB)

## Data Availability

The data analyzed in the current study are available from the corresponding author on reasonable request.
