# Supplementary material for: Differential accumulation of enterococci and arsenic in pelagic Sargassum and seagrass wrack on South Florida beaches
Source: Environ Monit Assess. 2026 Jan 14;198(2):126. doi: 10.1007/s10661-025-14888-5 (PMC12804244; doi:10.1007/s10661-025-14888-5)
Supplement: Supplementary file 1 — (DOCX 7.07 MB) [file 10661_2025_14888_MOESM1_ESM.docx]

**Supplemental Text for:**

**Differential Accumulation of Enterococci and Arsenic in Pelagic *Sargassum* and Seagrass Wrack on South Florida Beaches**

Afeefa A. Abdool-Ghany^1,2 *^, Ayaaz Amirali^1^, Rivka Reiner^1^, Sofia Hoffman^1^, Isabela Tavarez^1^, Matthew Roca^1^, Jiayu Li^3^, Helena Solo-Gabriele^1, *^

^1^ Department of Chemical, Environmental, and Materials Engineering, College of Engineering, University of Miami, Coral Gables, Florida, USA

^2^Brizaga, 2101 W Commercial Blvd, Fort Lauderdale, Florida, USA

^3^Department of Mechanical and Aerospace Engineering, College of Engineering, University of Miami, Coral Gables, Florida, USA

Email Addresses:

Afeefa Aleema Abdool-Ghany: aaa625@miami.edu

Ayaaz Amirali: a.amirali@umiami.edu

Rivka Reiner: rreiner@chenmoore.com

Sofia Hoffman: sph5917@psu.edu

Isabela Tavarez: ixt229@miami.edu

Matthew Roca: mxr1947@miami.edu

Jiayu Li: jiayuli@miami.edu

Helena Solo-Gabriele: hmsolo@miami.edu

For consideration in for potential publication in:

Environmental Monitoring and Assessment

Version Date: Otober 7, 2025

*Corresponding Author. Afeefa Abdool-Ghany: Tel. +1-954-298-4073, Email address: [aaa625@miami.com](mailto:aaa625@miami.com). Department of Chemical, Environmental, and Materials Engineering, College of Engineering, University of Miami, Coral Gables, Florida, USA

*Corresponding Author. Helena Solo-Gabriele: Tel. +1-305-989-9103, Email address: [hmsolo@miami.edu](mailto:hmsolo@miami.edu). Department of Chemical, Environmental, and Materials Engineering, College of Engineering, University of Miami, Coral Gables, Florida, USA


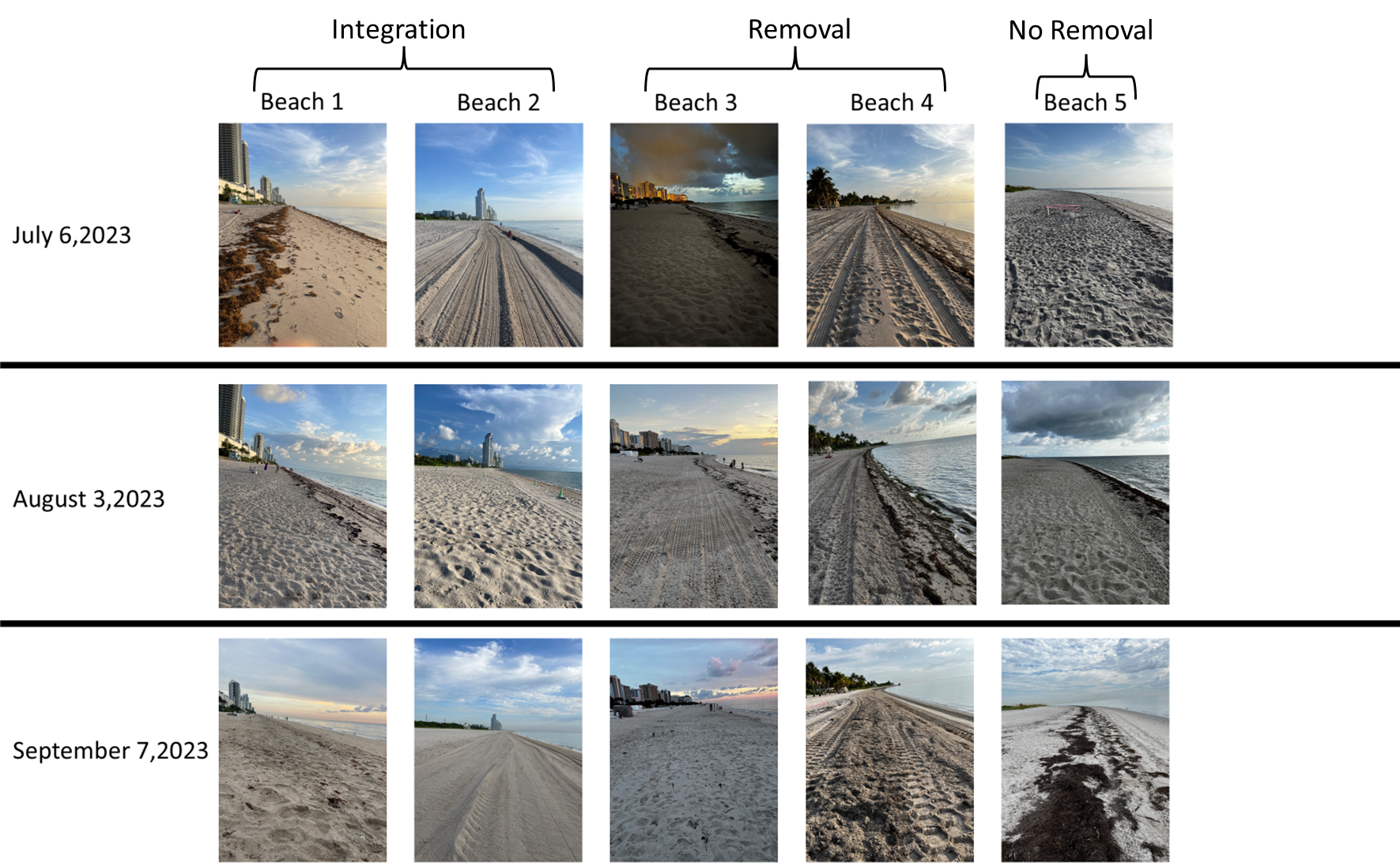


Figure S-1. Photos of five beach sites taken during sampling, depicting the environmental conditions at the time of sample collection. The images illustrate the physical state of the beaches under different management practices—Integration of beach wrack, Removal of beach wrack, and No Removal—highlighting the variability in wrack presence, sand conditions, and beach management approaches. Two sampling teams collected samples starting at their designated beaches at sunrise. Sampling commenced at Beach 1 at sunrise and then continued to Beach 2. Similarly sampling commenced at Beach 3 at sunrise and commenced to 4 and 5. Beach grooming usually started at beaches 1 through 4 shortly after sunrise and for this reason, beach grooming activities are more obvious for the second beaches (Beaches 2 and 4) visited by each team.


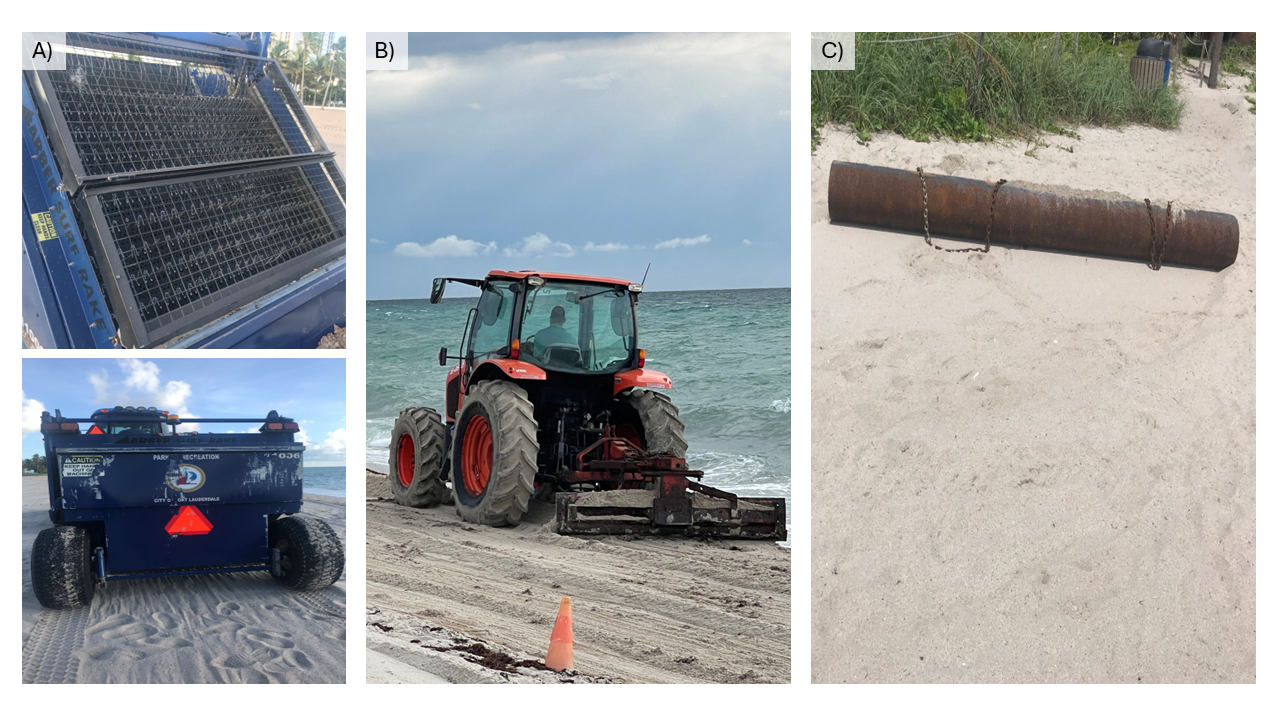


Figure S-2. Equipment used for wrack management on South Florida beaches. Wrack removal was conducted using a mechanical surf rake (Surf Rake by Barber) attached to a tractor, which lifts wrack onto a perforated conveyor belt that allows sand to fall back onto the beach while retaining the organic material for offsite disposal (A). Two integration methods were employed at different sites: the first involved a rear-mounted blader system that fragments the wrack and mixes it into the underlying sand (B), while the second used a pull bar composed of a large wooden or metal pole dragged behind a tractor to press the wrack into the sand along the deposition line (C).


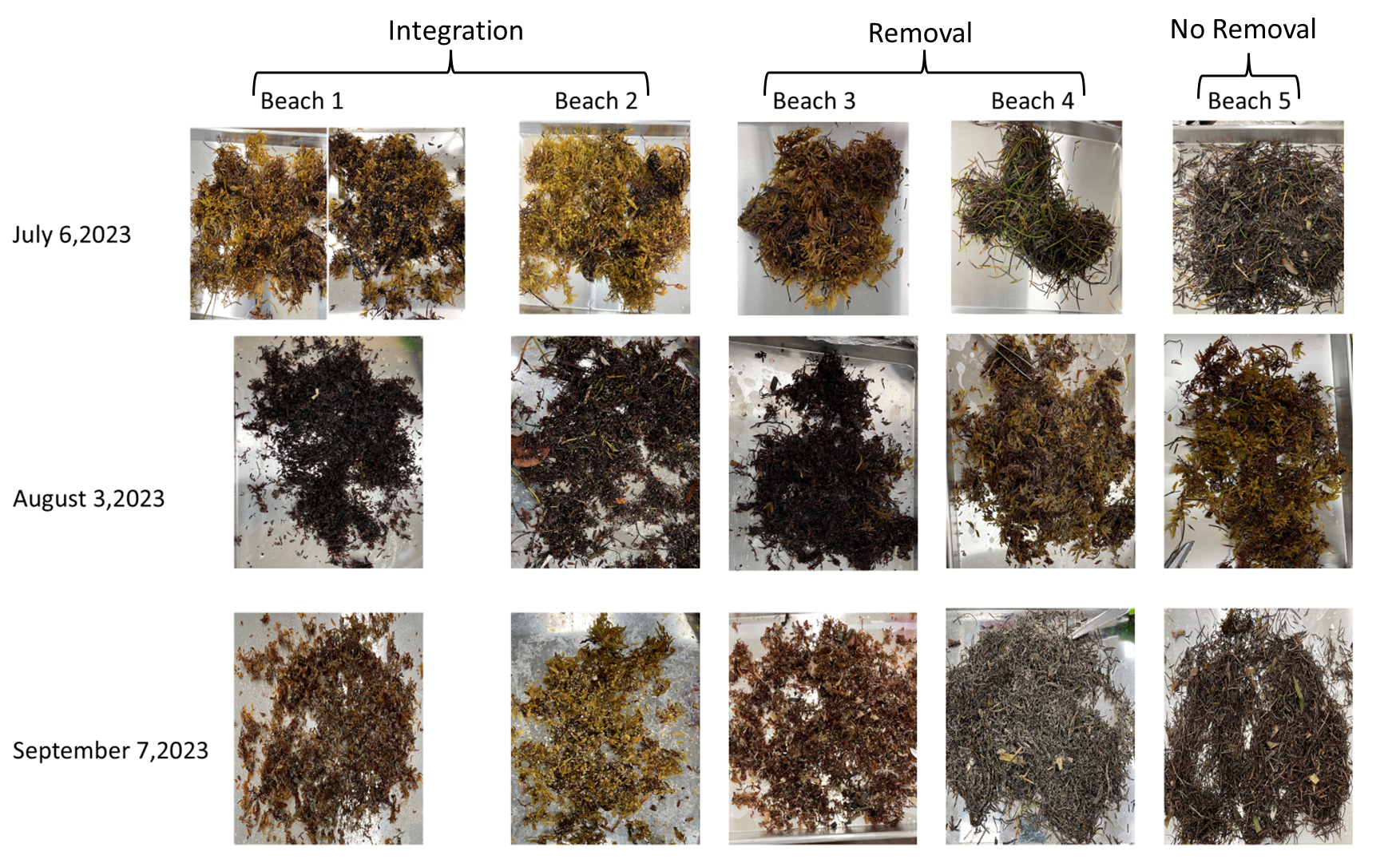


Figure S-3. Photos of wrack collected from three sampling periods at beaches practicing Integration, Removal, and No Removal management styles.

Table S-1. GPS coordinates of sampling locations.

| Site ID | GPS Coordinate | Arsenic Content (mg/kg) |
| --- | --- | --- |
| B1 – Hallandale Beach | 25°58'59.8"N 80°07'04.0"W | 35 |
| B2 – Haulover Beach | 25°54'19.0"N, 80°07'17.1"W | 32.7 |
| B3 – Miami Beach | 25°47'48.2"N, 80°7'32.2"W | 29.9 |
| B4 – Crandon Park Beach (Preserve) | 25°42'44.9"N, 80°9'5.2"W | 62.5 |
| B5- Cape Florida/Bill Baggs | 25°40'11.8"N, 80°9'12"W | 48.2 |

Table S-2. Ambient Environmental Data During Field Sample Collection

| Site ID | Environmental Parameter | July 6,  2023 | August 3, 2023 | September 7, 2023 |
| --- | --- | --- | --- | --- |
| B1 | Sampling Time | 7:04 am | 7:19 am | 7:18 am |
|  | General Weather Conditions | Sunny | Overcast | Overcast |
|  | Temperature of Water (°C) | 31.0 | 31.2 | 28.6 |
|  | Temperature of Sand (°C) | 27.3 | 28.4 | 26.1 |
|  | Temperature of Wrack (°C) | 27.3 | 28.4 | 25.4 |
|  | Air Temperature (°C) | 27 | 26 | 26 |
|  | Humidity (%) | 91 | 96.5 | 87.2 |
|  | Wave Height (cm) | 10.2 | 7.6 | NA^a^ |
|  | Wind (miles per hour) | Calm | Calm | Calm |
|  | Visibility (miles) | 10 | 10 | 10 |
|  | pH | 8.1 | 8.2 | 8.2 |
|  | Turbidity (ntu) | 6.1 | 0.8 | 2.5 |
|  | Salinity (psu) | 33.4 | 35.5 | 32.6 |
| B2 | Sampling Time | 7:52 am | 8:02 am | 8:07 am |
|  | General Weather Conditions | Sunny | Sunny | Overcast |
|  | Temperature of Water (°C) | 30.4 | 30.1 | NA |
|  | Temperature of Sand (°C) | 28.0 | 29.0 | 25.9 |
|  | Temperature of Wrack (°C) | NA | 28.9 | 25.7 |
|  | Air Temperature (°C) | 27 | 28 | 26 |
|  | Humidity (%) | 87.8 | 84.8 | 84.6 |
|  | Wave Height (cm) | 12.7 | 12.7 | NA |
|  | Wind (miles per hour) | Calm | Calm | 4 |
|  | Visibility (miles) | 10 | 10 | 10 |
|  | pH | 8.1 | 8.2 | 8.1 |
|  | Turbidity (ntu) | 1.4 | 3.2 | 0.5 |
|  | Salinity (psu) | 33.2 | 35.4 | 33.4 |
| B3 | Sampling Time | 6:03 am | 6:45 am | 7:00 am |
|  | General Weather Conditions | Overcast | Sunny | Sunny |
|  | Temperature of Water (°C) | 31.1 | 29.3 | 28.8 |
|  | Temperature of Sand (°C) | 26.0 | 26.5 | 24.8 |
|  | Temperature of Wrack (°C) | 24.3 | 26.6 | 26.5 |
|  | Air Temperature (°C) | 28 | 27 | 27 |
|  | Humidity (%) | 85.4 | 82.2 | 74.2 |
|  | Wave Height (cm) | 25.4 | 15.2 | 30.5 |
|  | Wind (miles per hour) | Calm | Calm | Calm |
|  | Visibility (miles) | 10 | 10 | 10 |
|  | pH | 8.1 | 8.1 | 8.0 |
|  | Turbidity (ntu) | 4.5 | 0.1 | 5.5 |
|  | Salinity (psu) | 34.1 | 35.5 | 33.4 |

Table S-2 (continued). Ambient Environmental Data During Field Sample Collection

| Site ID | Environmental Parameter | July 6,  2023 | August 3, 2023 | September 7, 2023 |
| --- | --- | --- | --- | --- |
| B4 | Sampling Time | 7:15 am | 7:51 am | 8:15 am |
|  | General Weather Conditions | Sunny | Sunny | Sunny |
|  | Temperature of Water (°C) | 31.0 | 29.4 | 29.2 |
|  | Temperature of Sand (°C) | 26.6 | 31.5 | 27.0 |
|  | Temperature of Wrack (°C) | 26.6 | 30.3 | 27.2 |
|  | Air Temperature (°C) | 28 | 27 | 28 |
|  | Humidity (%) | 85.4 | 84.7 | 71.7 |
|  | Wave Height (cm) | 2.5 | 7.7 | 7.6 |
|  | Wind (miles per hour) | Calm | Calm | Calm |
|  | Visibility (miles) | 10 | 10 | 10 |
|  | pH | 8.1 | 7.7 | 8.2 |
|  | Turbidity (ntu) | 7.4 | 15.2 | 2.7 |
|  | Salinity (psu) | 33.1 | 33.8 | 33.0 |
| B5 | Sampling Time | 8:36 am | 8:35 am | 8:59 am |
|  | General Weather Conditions | Sunny | Overcast | Sunny |
|  | Temperature of Water (°C) | 31.8 | 29.5 | 28.3 |
|  | Temperature of Sand (°C) | 30.5 | 28.6 | 27.1 |
|  | Temperature of Wrack (°C) | 30.1 | 28.4 | 29.3 |
|  | Air Temperature (°C) | 29 | 29 | 28 |
|  | Humidity (%) | 80.1 | 79.6 | 69.6 |
|  | Wave Height (cm) | 6.4 | 12.7 | 10.2 |
|  | Wind (miles per hour) | 3 | Calm | 3 |
|  | Visibility (miles) | 10 | 10 | 10 |
|  | pH | 8.1 | 8.2 | 7.7 |
|  | Turbidity (ntu) | 16.4 | 2.5 | 2.5 |
|  | Salinity (psu) | 31.9 | 34.1 | 32.0 |

^a^NA=Not Available

Table S-3. Raw data from the study, presenting the concentrations of bacteria and arsenic across various management styles—Integration, Removal, and No Removal. All water samples were collected in ankle deep water and are identified as “Ankle Water”. Sand identified as “Sand Under”, “Supratidal” and “Bladed”. Wrack identified as “*Sargassum*” and “Seagrass”.

| **Beach Identifier** | **Sample Date** | **Sample Type** | **Bacteria Concentration (CFU/100 mL or dry g)**^a^ | **Arsenic Concentration (mg/kg)** | **Management style** | **Moisture content** |
| --- | --- | --- | --- | --- | --- | --- |
| 1 | 7/6/2023 | Ankle Water | 80.0 | ND^b^ | Integration | NA^c^ |
| 1 | 7/6/2023 | Sand Under | 36.1 | 3.60 | Integration | 0.0923 |
| 1 | 7/6/2023 | *Sargassum* | 875 | 33.25 | Integration | 0.7153 |
| 1 | 7/6/2023 | Supratidal | 344 | 4.13 | Integration | 0.0031 |
| 1 | 8/3/2023 | Ankle Water | 91.0 | ND | Integration | NA |
| 1 | 8/3/2023 | Sand Under | 353 | 4.12 | Integration | 0.0392 |
| 1 | 8/3/2023 | *Sargassum* | 5,292 | 9.99 | Integration | 0.5269 |
| 1 | 8/3/2023 | Supratidal | 72.0 | 4.41 | Integration | 0.0164 |
| 1 | 9/7/2023 | Ankle Water | 74.0 | ND | Integration | NA |
| 1 | 9/7/2023 | Sand Under | 27.0 | 4.92 | Integration | 0.0208 |
| 1 | 9/7/2023 | *Sargassum* | 57.0 | 61.70 | Integration | 0.2292 |
| 1 | 9/7/2023 | Supratidal | 248 | 4.68 | Integration | 0.0067 |
| 2 | 7/6/2023 | Ankle Water | 16.0 | ND | Integration | NA |
| 2 | 7/6/2023 | Bladed Sand | 196 | 2.02 | Integration | 0.0264 |
| 2 | 7/6/2023 | Sand Under | 2.1 | 1.65 | Integration | 0.0436 |
| 2 | 7/6/2023 | *Sargassum* | 51.6 | 23.00 | Integration | 0.6998 |
| 2 | 7/6/2023 | Supratidal | 69.0 | 1.30 | Integration | 0.0010 |
| 2 | 8/3/2023 | Ankle Water | 244 | ND | Integration | NA |
| 2 | 8/3/2023 | Bladed Sand | 625 | 1.73 | Integration | 0.0337 |
| 2 | 8/3/2023 | Sand Under | 230 | 1.89 | Integration | 0.0311 |
| 2 | 8/3/2023 | *Sargassum* | 9,642 | 10.70 | Integration | 0.6667 |
| 2 | 8/3/2023 | Supratidal | 304 | 1.65 | Integration | 0.0023 |
| 2 | 9/7/2023 | Ankle Water | 118 | ND | Integration | NA |
| 2 | 9/7/2023 | Bladed Sand | 8.8 | 1.54 | Integration | 0.0349 |
| 2 | 9/7/2023 | Sand Under | 0.6 | 1.77 | Integration | 0.0203 |
| 2 | 9/7/2023 | *Sargassum* | 13.9 | 64.30 | Integration | 0.4485 |
| 2 | 9/7/2023 | Supratidal | 100 | 1.85 | Integration | 0.0012 |
| 3 | 7/6/2023 | Ankle Water | 50.0 | ND | Removed | NA |
| 3 | 7/6/2023 | Sand Under | 16.6 | 1.70 | Removed | 0.0337 |
| 3 | 7/6/2023 | *Sargassum* | 15.9 | 43.70 | Removed | 0.6845 |
| 3 | 7/6/2023 | Supratidal | 38.0 | 1.55 | Removed | 0.0019 |
| 3 | 8/3/2023 | Ankle Water | 29.0 | ND | Removed | NA |
| 3 | 8/3/2023 | Sand Under | 648 | 1.08 | Removed | 0.0341 |
| 3 | 8/3/2023 | *Sargassum* | 8,594 | 10.90 | Removed | 0.6891 |
| 3 | 8/3/2023 | Supratidal | 267 | 1.18 | Removed | 0.0025 |

Table S-3. continued.

| **Beach Identifier** | **Sample Date** | **Sample Type** | **Bacteria Concentration (CFU/100 mL or dry g)** | **Arsenic Concentration (mg/kg)** | **Management style** | **Moisture content** |
| --- | --- | --- | --- | --- | --- | --- |
| 3 | 9/7/2023 | Ankle Water | 15.0 | ND | Removed | NA |
| 3 | 9/7/2023 | Sand Under | 32.5 | 0.58 | Removed | 0.0068 |
| 3 | 9/7/2023 | *Sargassum* | 1662 | 35.10 | Removed | 0.2859 |
| 3 | 9/7/2023 | Supratidal | 121 | 2.26 | Removed | 0.0030 |
| 4 | 7/6/2023 | Ankle Water | 42.0 | ND | Removed | NA |
| 4 | 7/6/2023 | Sand Under | 259 | 2.03 | Removed | 0.0597 |
| 4 | 7/6/2023 | Seagrass | 2,435 | 1.55 | Removed | 0.8112 |
| 4 | 7/6/2023 | Supratidal | 94.0 | 2.19 | Removed | 0.0038 |
| 4 | 8/3/2023 | Ankle Water | 145 | ND | Removed | NA |
| 4 | 8/3/2023 | Sand Under | 476 | 2.45 | Removed | 0.0670 |
| 4 | 8/3/2023 | *Sargassum* | 2,742 | 62.50 | Removed | 0.5816 |
| 4 | 8/3/2023 | Supratidal | 38.8 | 1.94 | Removed | 0.0088 |
| 4 | 9/7/2023 | Ankle Water | 28.0 | ND | Removed | NA |
| 4 | 9/7/2023 | Sand Under | 1,158 | 1.92 | Removed | 0.0445 |
| 4 | 9/7/2023 | Seagrass | 2966 | 2.03 | Removed | 0.5083 |
| 4 | 9/7/2023 | Supratidal | 586 | 2.33 | Removed | 0.002 |
| 5 | 7/6/2023 | Ankle Water | 7.0 | ND | No Removal | NA |
| 5 | 7/6/2023 | Sand Under | 54.2 | 1.29 | No Removal | 0.0428 |
| 5 | 7/6/2023 | Seagrass | 2,973 | 1.77 | No Removal | 0.5878 |
| 5 | 7/6/2023 | Supratidal | 429 | 1.42 | No Removal | 0.0053 |
| 5 | 8/3/2023 | Ankle Water | 21.0 | ND | No Removal | NA |
| 5 | 8/3/2023 | Sand Under | 10.2 | 1.35 | No Removal | 0.1313 |
| 5 | 8/3/2023 | *Sargassum* | 68.7 | 48.20 | No Removal | 0.7613 |
| 5 | 8/3/2023 | Supratidal | 96.0 | 1.61 | No Removal | 0.0046 |
| 5 | 9/7/2023 | Ankle Water | 79.0 | ND | No Removal | NA |
| 5 | 9/7/2023 | Sand Under | 18.3 | 1.06 | No Removal | 0.0452 |
| 5 | 9/7/2023 | Seagrass | 3,554 | 2.18 | No Removal | 0.5399 |
| 5 | 9/7/2023 | Supratidal | 168 | 1.62 | No Removal | 0.0019 |

^a^“Ankle water” has units of CFU/100 mL. All other sample types have units of CFU/dry_gram,

^b^ND=Not Detected. Detection limit of 30 µg/L

^c^NA=Not applicable
